# Bapineuzumab for mild to moderate Alzheimer’s disease: a meta-analysis of randomized controlled trials

**DOI:** 10.1186/s12883-017-0850-1

**Published:** 2017-04-04

**Authors:** Abdelrahman Ibrahim Abushouk, Ahmed Elmaraezy, Amro Aglan, Reham Salama, Samar Fouda, Rana Fouda, Ammar M. AlSafadi

**Affiliations:** 1 Faculty of Medicine, Ain Shams University, Cairo, Egypt; 2 NovaMed Medical Research Association, Cairo, Egypt; 3 Faculty of Medicine, Al-Azhar University, Cairo, Egypt; 4 Faculty of Medicine, Tanta University, Tanta, Egypt; 5 Faculty of Medicine, Benha University, Qaluobia, Egypt; 6 Faculty of Medicine, Zagazig University, Elsharkia, Egypt; 7 Faculty of Medicine, Damascus University, Damascus, Syria

**Keywords:** Bapineuzumab, Passive immunotherapy, Alzheimer’s disease, Dementia

## Abstract

**Background:**

Alzheimer’s disease (AD) is a globally prevalent neurodegenerative condition, clinically characterized by progressive memory loss and gradual impairment of cognitive functions. Bapineuzumab is a fully humanized monoclonal antibody that binds to neurotoxic amyloid proteins in the brain, enhancing their clearance. We performed this systematic review and meta-analysis to evaluate the safety and efficacy of bapineuzumab in patients with mild to moderate Alzheimer’s disease.

**Methods:**

We performed a web-based literature search of PubMed, Ovid, EBSCO, Scopus, Embase, Cochrane CENTRAL, and web of science using the relevant keywords. Data were extracted from eligible records and pooled as mean difference (MD) or risk ratio (RR) values with their 95% confidence interval (CI), using Review Manager software (﻿version 5.3 for windows). Heterogeneity was measured by Chi-square and I-square tests.

**Result:**

The pooled effect estimate from six randomized clinical trials (*n* = 2380) showed that bapineuzumab significantly reduced the cerebrospinal fluid concentration of phosphorylated tau proteins (Standardized MD = −5.53, 95% CI [−8.29, −2.76]). However, the bapineuzumab group was not superior to the placebo group in terms of change from baseline in Alzheimer’s disease assessment scale (ADAS)-Cog11 (MD = 0.14, 95% CI [−0.72, 0.99]), disability assessment for dementia (DAD) scale (MD = 1.35, 95% CI [−1.74, 4.43]), and mini-mental state examination (MMSE) scores (MD = 0.08, 95% CI [−0.31, 0.47]). Regarding safety, bapineuzumab increased the risk of serious treatment-emergent adverse events (RR = 1.18, 95% CI [1.02, 1.37]) and cerebral vasogenic edema (RR = 40.88, 95% CI [11.94, 135.95]). All bapineuzumab doses (0.15, 0.5, 1, and 2 mg/kg) were similar to placebo in terms of change from baseline in ADAS-cog11, DAD, and MMSE scores, except for the 0.15 mg/kg dose, which caused a significant worsening on the ADAS-cog11 scale (MD = 5.6, 95% CI [0.22, 10.98]).

**Conclusions:**

Considering the lack of clinical efficacy, combined with the significant association with serious adverse events, bapineuzumab should not be used to treat patients with mild to moderate AD. Future studies should investigate the effect of combining bapineuzumab with other therapeutic strategies and reevaluate the efficacy of targeting amyloid β proteins in AD therapy.

**Electronic supplementary material:**

The online version of this article (doi:10.1186/s12883-017-0850-1) contains supplementary material, which is available to authorized users.

## Background

Alzheimer’s Disease (AD) is a neurodegenerative condition, clinically characterized by progressive memory loss and gradual impairment of cognitive functions [1]. The pathological hallmarks of the disease include cerebral neuronal loss, cerebral plaques due to accumulation of extracellular amyloid β (Aβ) proteins, and intraneuronal neurofibrillary tangles [2–4]. The current annual incidence of AD is 1275 new cases per 100,000 patients [5] and the prevalence is expected to reach more than 140 million patients in 2050 [6]. Current therapeutic strategies only aim at improving the symptoms by improving neurotransmitter levels in the surviving neuronal circuitry [7].

Babineuzumab (AAB-001) is a fully - humanized, N-terminal specific anti-Aβ monoclonal antibody, which binds to neurotoxic amyloid proteins in the brain, enhancing their clearance [8]. Preclinical trials have shown that passive immunotherapy with monoclonal antibodies was associated with a significant reduction of Aβ protein levels in the brain and memory improvement in transgenic mice with Aβ proteins overproduction [8–12]. Furthermore, phase II clinical trials have shown that bapineuzumab can reduce the load of amyloid proteins on positron emission tomography (PET) and the concentration of phosphorylated tau proteins in the cerebrospinal fluid [13, 14].

Within the past few years, several phase II and phase III clinical trials have investigated the role of bapineuzumab in improving the clinical and biomarker outcomes of AD [13–17]. The purpose of this systematic review and meta-analysis is to synthesize evidence from published, randomized, controlled trials regarding the safety and efficacy of bapineuzumab in the treatment of patients with mild to moderate AD.

## Methods

We followed the PRISMA statement guidelines during the preparation of this systematic review and meta-analysis. Moreover, all steps were performed in a strict accordance to the Cochrane handbook of systematic reviews of interventions [18].

### Literature search strategy

We searched for published, randomized, controlled trials in medical electronic databases including: PubMed, Ovid, EBSCO, Scopus, Embase, Cochrane central register of clinical trials (CENTRAL), and web of science through April 2016, using the following query: “Bapineuzumab OR AAB-001 AND Alzheimer OR Dementia”. We also checked the clinical trial registry (Clinicaltrials.gov) for additional ongoing and unpublished studies. No language or time restrictions were imposed. Furthermore, we hand-searched the reference list of included studies for any missed trials.

### Eligibility criteria and study selection

We used the following inclusion criteria:

1. Study design: randomized controlled trials comparing bapineuzumab with placebo.
2. Intervention:
  ➢ Drug: Bapineuzumab
  ➢ Dose: 0.15, 0.5, 1, or 2 mg/kg. Other doses were not adequately reported in included studies; therefore, there were not eligible for quantitative analysis.
  ➢ Preparation/route of administration: Intravenous infusion.
3. Comparator: placebo (control group).
4. Population: Patients with mild to moderate AD (MMSE score between 14 and 26 and Rosen Modified Hachinski Ischemic score < 4). Patients were excluded if they had another clinically significant neurological disease.
5. Outcomes: Efficacy endpoints included clinical and key biomarker outcomes. Safety endpoints included commonly reported adverse events in the analyzed trials.

We excluded observational studies, animal studies, non-randomized trials, studies with unreliable data extraction, thesis, and conference abstracts. Two independent authors (AA, RS) screened the title and abstract of retrieved records for relevance to the review subject. Full texts of potentially relevant studies were retrieved and reviewed for eligibility to meta-analysis.

### Data extraction

Two authors (AIA, RF) extracted the data independently using a formatted data extraction sheet. A consensus between the review authors was obtained to prevent any misinterpretation of extracted data and any conflicts were resolved upon the opinion of a third reviewer (AE). The extracted data included the following: 1) criteria of study design, 2) characteristics of enrolled patients, 3) study outcomes including:

A. Efficacy outcomes:
  ➢ Clinical outcomes included change from baseline scores in the 11-item cognitive subscale of the Alzheimer’s Disease Assessment Scale (ADAS-cog11: with scores between 0 and 70; higher scores indicate greater impairment [19]), the Disability Assessment for Dementia (DAD: scale with scores between 0 and 100; higher scores indicate less impairment [20]), clinical dementia rating scale-sum of boxes (CDR-SOB: with scores between 0 and 18; higher scores indicate greater impairment [21]), neuropsychological battery test score (which is scored on a standardized z scale; higher scores indicate less impairment [22]), mini-mental state examination (MMSE), and dependence scale (with scores between 0 and 15; higher scores indicate greater assistance requirements [23]).
  ➢ Key biomarker outcomes included brain amyloid burden, measured by Pittsburgh compound B- Positron emission tomography (PIB-PET) and calculated as the average standardized uptake value ratio (SUVR) of cortical regions, cerebrospinal fluid phosphorylated-tau protein concentration (pg/ml), and the annual rate of brain volume loss, measured by volumetric MRI.
B. Safety outcomes included the frequency of serious treatment emergent adverse events (TEAEs), fatal adverse events, amyloid related imaging abnormalities (vasogenic edema), delirium, headache, and convulsions.

Data for continuous outcomes (efficacy endpoints) were extracted as change score (mean difference or change from baseline to the treatment endpoint [week 78]) and standard deviation, while data for dichotomous outcomes (safety endpoints) were extracted as the number of events in each study group, compared to the total number of enrolled patients within that group. When the standard deviation (SD) of mean change from baseline was missing, it was calculated from the standard error (SE) or 95% confidence interval (CI) according to Altman equations [24].

### Risk of bias assessment

Two authors (AIA, SF) independently assessed the risk of bias in included studies, in accordance with the Cochrane handbook of systematic reviews of interventions (5.1.0). Due to the small number of included studies, publication bias could not be assessed using Begg’s funnel-plot-based methods or Egger’s regression test [25].

### Data synthesis

Changes in efficacy outcomes were pooled as mean difference (MD) or standardized mean difference (SMD) values and the frequency of adverse events was pooled as risk ratio (RR) values with a confidence interval of 95% in a meta-analysis model. Statistical analysis was conducted by Review Manager (RevMan) software (version 5.3 for windows). The results were considered statistically significant if the *p* value was less than 0.05. Heterogeneity among included studies was measured by the Chi-Square test and the I-Square test was used to quantify its extent. In case of significant heterogeneity (Chi-Square *p* < 0.1 or I^2^ > 50%), the analysis was conducted under the random effects model; otherwise, a fixed effect model was used.

## Results

**Fig. 1:**
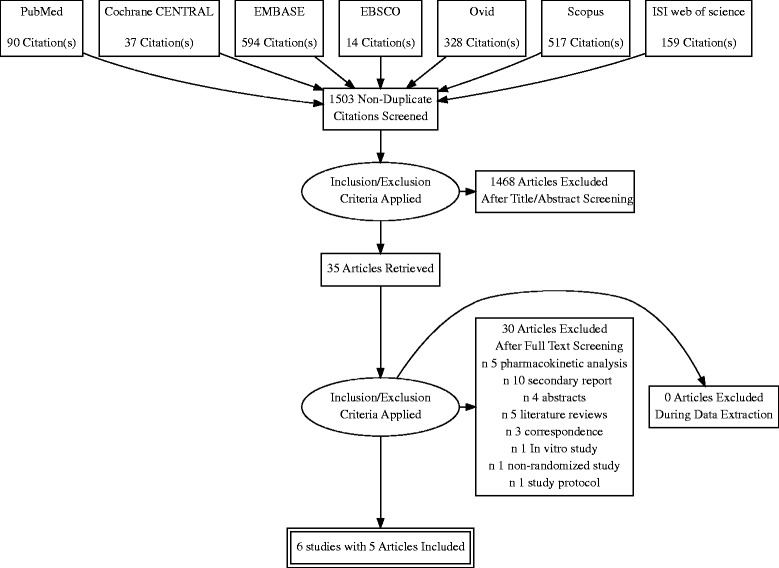
PRISMA flow diagram of studies’ screening and selection

Our search retrieved 1503 unique records. Thirty five full text articles were screened for eligibility, from which 30 articles were excluded. Six unique studies (five reports with 2380 patients) were included in the final analysis. (See PRISMA flow diagram; Fig. 1). Reasons for study exclusion are shown in Additional file 1. A summary of included studies and their primary results are shown in Table 1 and baseline characteristics of their enrolled patients are shown in Table 2.

**Table 1:** Shows a summary of the design and main findings of included studies

Study ID	Study Design	Population	Sample size in each group		Finding
			Bapineuzumab	Placebo	
Salloway et al., 2009 [13]	- Phase II, randomized in a ratio of 8:7, double- blinded. - Patients received 6 infusions of bapineuzumab or placebo, 13 weeks apart. Final assessment occurred at 78 weeks.	• Patients aged from 50 to 85 years. • Met criteria for AD and had MRI findings, consistent with AD. • Mini-Mental State Examination (MMSE) score of 16 to 26. • Rosen Modified Hachinski Ischemic score < 4. • Participants were excluded from the study if they had a clinically significant neurological (other than AD) or systemic disease.	124 patients were allocated in 1 of 4 sequential dose cohorts (0.15, 0.5, 1.0, or 2.0 mg/kg).	110 patients were allocated to Placebo group.	No significant treatment differences were found in the primary efficacy analysis. However, exploratory analyses showed potential differences on cognitive and functional outcomes in study completers and APOE- 4 non-carriers.
Salloway et al., 2014 (Carriers) [16]	- Phase IIIa, randomized in a ratio of 3:2, double blinded. - Patients received 6 infusions of bapineuzumab or placebo, 13 weeks apart. Final assessment occurred at 78 weeks.	• Patients aged from 50 to 88 years. • Met criteria for AD and had MRI findings, consistent with AD. • Carriers of the apolipoprotein E (*APOE*) *ε4* allele • Mini–Mental State Examination (MMSE) of 16 to 26. • Score on the Hachinski Ischemic scale of 4 or lower. • Participants were excluded from the study if they had a clinically significant neurological (other than AD) or systemic disease.	658 patients were allocated to bapineuzumab 0.5 mg per kg group.	432 patients were allocated to placebo group.	No differences between bapineuzumab and placebo in terms of change from baseline in the ADAS-cog11 and DAD scores (*P* = 0.80 and 0.34 respectively). Therefore, Bapineuzumab did not improve the clinical outcomes in AD patients.
Salloway et al., 2014 (Non-carriers) [16]	- Phase IIIb, randomized 3:3:4, double blinded. - Patients received 6 infusions of bapineuzumab or placebo, 13 weeks apart. Final assessment occurred at 78 weeks.	• Patients aged from 50 to 88 years. • Met criteria for AD and had MRI findings, consistent with AD. • Non-Carriers of the apolipoprotein E (*APOE*) *ε4* allele • Mini–Mental State Examination (MMSE) of 16 to 26. • Score on the Hachinski Ischemic scale of 4 or lower. • Participants were excluded from the study if they had a clinically significant neurological (other than AD) or systemic disease.	621 patients were allocated to bapineuzumab groups. - Bapineuzumab 0.5 mg per kg: 337 - Bapineuzumab 1.0 mg per kg: 307	524 patients were allocated to placebo group.	No differences were found between bapineuzumab (Both doses) and placebo groups in terms of change from baseline in the ADAS-cog11 and DAD scores (*P* = 0.64 and 0.07 respectively). Therefore, bapineuzumab did not improve the clinical outcomes in patients with AD.
Black et al., 2010 [15]	- Phase II, randomized, third-party unblinded. - Patients received bapineuzumab or placebo with dose escalation every 10 weeks. Final assessment occurred at 52 weeks.	• Patients aged from 50 to 88 years. • Met criteria for AD and had MRI findings, consistent with AD. • Mini-Mental State Examination (MMSE) score of 14 to 26. • Rosen Modified Hachinski Ischemic score ≤ 4. • Participants were excluded from the study if they had a clinically significant neurological (other than AD) or systemic disease.	22 patients were allocated to bapineuzumab groups. - Bapineuzumab 0.5 mg/kg: 6 - Bapineuzumab 1.5 mg/kg: 6 - Bapineuzumab 5 mg/kg: 10	8 patients were allocated to placebo group.	MMSE scores improved at lower doses of bapineuzumab (0.5 and 1.5 mg/kg) compared to placebo, but not with the highest dose (5 mg/kg). Moreover, The 5 mg/kg dose was significantly associated with MRI amyloid abnormalities that resolved over time.
Arai et al., 2015 [17]	- Phase I, randomized, double- blinded. - Patients received one infusion of the study drug or placebo. Final assessment occurred at 52 weeks.	• Patients aged from 50 to 85 years. • Met criteria for AD and had MRI findings, consistent with AD. • Mini-Mental State Examination (MMSE) score of 14 to 26. • Rosen Modified Hachinski Ischemic score ≤ 4. • Participants were excluded from the study if they had a clinically significant neurological (other than AD) or systemic disease.	24 patients were equally allocated to 4 dose cohorts (0.15, 0.5, 1, or 2 mg/kg).	8 patients were allocated to placebo group.	Plasma β-amyloid levels increased with increasing doses of bapineuzumab. Bapineuzumab was well tolerated at all doses in Japanese patients with mild to moderate AD.
Rinne et al., 2010 [14]	- Phase II, randomized, double- blinded. - Patients received 6 infusions of bapineuzumab or placebo, 13 weeks apart. Final assessment occurred at 78 weeks.	• Patients aged from 50 to 85 years. • Met criteria for AD and had MRI findings, consistent with AD. • Mini-Mental State Examination (MMSE) score of 18 to 26. • Rosen Modified Hachinski Ischemic score ≤ 4. • Participants had amyloid-B loads in the range expected for patients with Alzheimer’s disease, defined as 11C–PiB PET retention ratios relative to the cerebellum of 1 to 5 or more in at least three brain regions among the anterior cingulate, posterior cingulate, frontal, temporal, and parietal cortices. • Participants were excluded from the study if they had a clinically significant neurological (other than AD) or systemic disease.	19 patients were allocated to 1 of 3 sequential dose cohorts (0.5, 1.0, 2.0 mg/kg).	7 patients were allocated to placebo group.	Treatment with bapineuzumab for 78 weeks reduced cortical carbon-11-labelled Pittsburgh compound B (C-PiB) retention, compared to placebo. Adverse events were typically mild to moderate in severity and transient in duration.

**Table 2:** Shows baseline characteristics of enrolled patients in included studies

Study ID	Drug	N	Age (Mean ± SD)	Sex (male) n (%)	Race (White) n	MMSE score (Mean ± SD)	AChEI or memantineuse, n (%)	*APOE ε4* status carrier no. (%)	ADAS-cog score (Mean ± SD)	DAD score (Mean ± SD)
SSalloway et al., 2009 [13]	Placebo	107	67.9 ± 8.8	43 (40.2)	102 (95.3)	20.7 ± 3.1	103 (96.3)	74 (69.8)	…..	….
	Bapineuzumab	122	70.1 ± 9.6	661 (50.0)	118 (96.7)	20.9 ± 3.2	116 (95.1)	72 (60.5)	…..	…..
Black et al., 2010 [15]	Placebo	8	69.8 ± 10.7	11 (12.5)	5 (62.5)	20.8	.....	.....	.....	.....
	Bapineuzumab 0.5 mg/kg	6	74.67 ± 5.65	33 (50)	4 (66.67)	21.8	.....	.....	.....	.....
Rinne et al., 2010 [14]	Placebo	7	70 ± 8·81	33 (43)	7 (100)	22·29 ± 2·69	7 (100)	5 (71)	19·19 ± 5·27	93·78 ± 8·24
	Bapineuzumab	19	67·26 ± 8·60	311 (57.9)	19 (100)	21·00 ± 2·33	19 (100)	12 (63)	22·26 ± 7·65	84·38 ± 11·95
Salloway et al., 2014 (Carrier) [16]	Placebo	432	72.3 ± 8.4	190 (44)	420 (97.2)	20.7 ± 3.2	400 (92.6)	432 (100)	23.9 ± 9.5	79.4 ± 18.9
	Bapineuzumab 0.5 mg/kg	658	72.0 ± 8.0	300 (45.6)	624 (94.8)	20.8 ± 3.1	606 (92.1)	658 (100)	23.5 ± 9.4	80.9 ± 17.3
Salloway et al., 2014 (Non-Carrier) [16]	Placebo	493	71.9 ± 10.1	245 (49.7)	469 (95.1)	21.2 ± 3.2	442 (89.7)	493 (100)	23.5 ± 9.4	80.5 ± 19.2
	Bapineuzumab 0.5 mg/kg	314	73.1 ± 9.3	149 (47.5)	298 (94.9)	21.2 ± 3.4	281 (89.5)	314 (100)	22.4 ± 9.7	80.0 ± 18.1
	Bapineuzumab 1 mg/kg	307	73.5 ± 9.1	132 (42.9)	292 (95.1)	21.2 ± 3.3	278 (90.6)	307 (100)	22.2 ± 10	80.4 ± 18.8
Arai et al., 2015 [17]	Placebo	8	68.8 ± 8.9	4 (50)	.....	20.6 ± 3.0	.....	.....	.....	.....
	Bapineuzumab 0.5 mg/kg	6	72.2 ± 8.4	5 (83.3)	.....	21.0 ± 3.6	.....	.....	.....	.....
	Bapineuzumab 1 mg/kg	6	72.2 ± 10.9	3 (50)	.....	21.0 ± 4.6	.....	.....	.....	.....

Abbreviations: *AChEI* Acetyl-Choline Esterase Inhibitor, *ADAS-Cog 11* Alzheimer disease assessment scale - Cognitive subscale 11 items, *DAD* Disability assessment scale, *MMSE* Mini-Mental State Examination

**Fig. 2:**
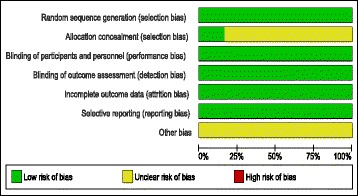
Risk of bias summary generated by RevMan software

The risk of bias in included studies was low according to the Cochrane risk of bias assessment tool. A summary of risk of bias assessment domains for included studies is shown in Fig. 2 and the authors’ judgments with justifications are shown in Additional file 2.

**Fig. 3:**
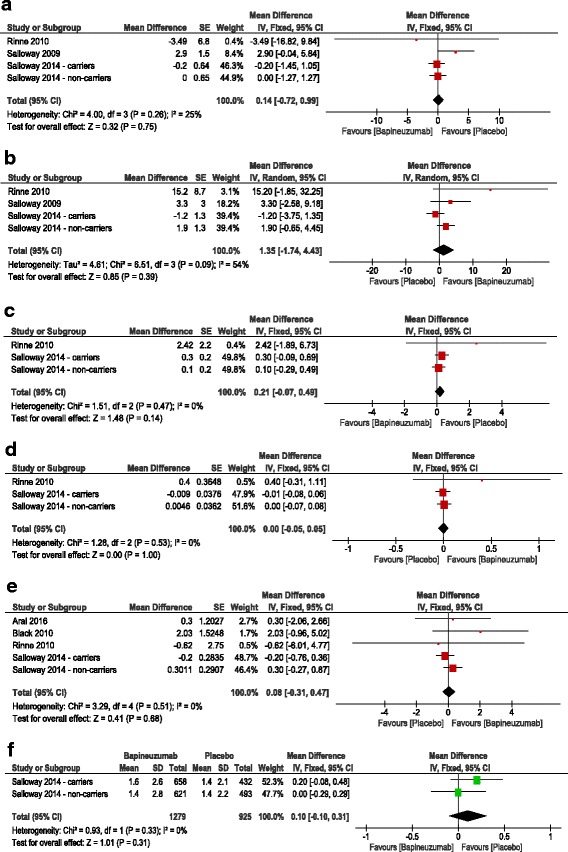
*Forest plot* of mean difference (MD) in **a** Alzheimer disease assessment scale - Cognitive subscale 11 items, **b** Disability assessment for dementia, **c** Clinical dementia rating scale – Sum of boxes, **d** Neuropsychological Battery test Score, **e** Mini Mental State Examination, and **f** Dependence scale score

I. Efficacy endpoints:

A. Clinical outcomes:

1. Alzheimer’s Disease Assessment Scale- Cognitive subscale score:
  The pooled effect size showed no significant difference between bapineuzumab and placebo groups in terms of change in ADAS-cog11 score from baseline to the treatment endpoint [week 78] (MD = 0.14, 95% CI [−0.72, 0.99], *p* = 0.75); Fig. 3a. Pooled studies were homogenous (I^2^ = 25%, *p* = 0.26).
2. Disability assessment for dementia score:
  The pooled effect size showed no significant difference between bapineuzumab and placebo groups in terms of change in DAD score from baseline to the treatment endpoint [week 78] (MD = 1.35, 95% CI [−1.74, 4.43], *p* = 0.39); Fig. 3b. Pooled studies were heterogenous (I^2^ = 54%, *p* = 0.09); therefore, the analysis was performed under the random effects model.
3. Clinical Dementia Rating Scale – Sum of boxes (CDR-SOB):
  The pooled effect size showed no significant difference between bapineuzumab and placebo groups in terms of change in CDR-SOB score from baseline to the treatment endpoint [week 78] (MD = 0.21, 95% CI [−0.07, 0.49], *p* = 0.14); Fig. 3c. Pooled studies were homogenous (I^2^ = 0%, *p* = 0.47).
4. Neuropsychological Battery test Score:
  The pooled effect size showed no significant difference between bapineuzumab and placebo groups in terms of change in neuropsychological battery test score from baseline to the treatment endpoint [week 78] (MD = 0.0, 95% CI [−0.05, 0.05], *p* = 1); Fig. 3d. Pooled studies were homogenous (I^2^ = 0%, *p* = 0.53).
5. Mini Mental State Examination (MMSE):
  The pooled effect size showed no significant difference between bapineuzumab and placebo groups in terms of change in MMSE score from baseline to the treatment endpoint [week 78] (MD = 0.08, 95% CI [−0.31, 0.47], *p* = 0.68); Fig. 3e. Pooled studies were homogenous (I^2^ = 0%, *p* = 0.51).
6. Dependence scale score:
  The pooled effect size showed no significant difference between bapineuzumab and placebo groups in terms of change in the dependence test score from baseline to the treatment endpoint [week 78] (MD = 0.10, 95% CI [−0.10, 0.31], *p* = 0.31); Fig. 3f. Pooled studies were homogenous (I^2^ = 0%, *p* = 0.33).

**Fig. 4:**
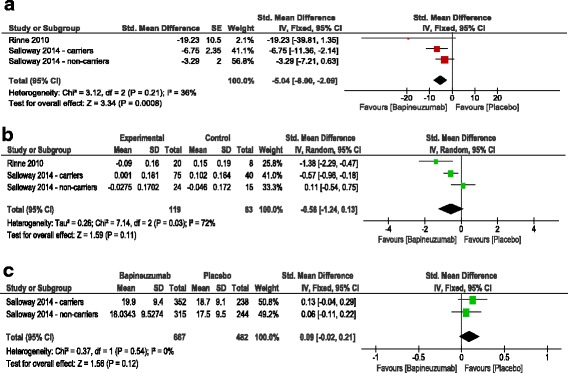
*Forest plot* of mean difference (MD) in **a** CSF phosphorylated tau protein concentration, **b** Standardized uptake value ratio, measured by PIB-PET, and **c** MRI whole-brain volume measurement

B. Key biomarker outcomes:

1. CSF Phosphorylated tau concentration:
  The overall effect estimate showed that bapineuzumab significantly reduced CSF tau-p concentrations at treatment endpoint [week 78], compared to placebo (SMD = −5.04, 95% CI [−8, −2.09], *p* = 0.0008); Fig. 4a. Pooled studies were homogenous (I^2^ = 24%, *p* = 0.26).
2. SUVR Measured by PIB-PET:
  The overall effect estimate showed no significant difference between bapineuzumab and placebo groups in terms of SVUR change from baseline to the treatment endpoint [week 78] (SMD = −0.56, 95% CI [−1.24, 0.13], *p* = 0.11); Fig. 4b. Pooled studies were heterogenous (I^2^ = 72%, *p* = 0.03); therefore, the analysis was performed under the random effects model.
3. MRI whole-brain volume measurement:
  The overall effect estimate showed no significant difference between bapineuzumab and placebo groups in terms of change of whole brain volume measurement from baseline to the treatment endpoint [week 78] (SMD = 0.09, 95% CI [−0.02, 0.21], *p* = 0.12); Fig. 4c. Pooled studies were homogenous (I^2^ = 0%, *p* = 0.54).

**Fig. 5:**
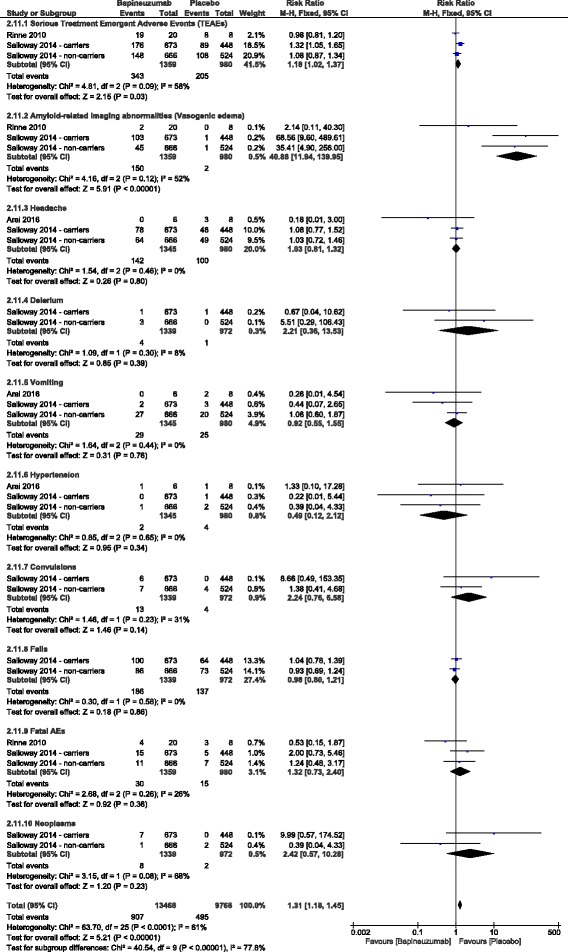
Forest plots of risk ratio (RR) of adverse events

**Fig. 6:**
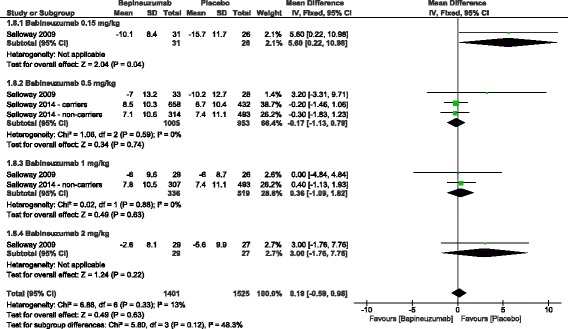
Stratification analysis of different bapineuzumab doses in terms of their effect on ADAS-Cog 11

II. Safety endpoints:
  The total incidence of adverse events was significantly higher in the bapineuzumab group, compared to the placebo group (RR = 1.31, 95% CI [1.18, 1.45], *p* < 0.00001). In terms of individual adverse events, the incidence of serious TEAEs (RR = 1.18, 95% CI [1.02, 1.37], *p* = 0.03) and amyloid-related imaging abnormalities (vasogenic edema) (RR = 40.88, 95% CI [11.94, 139.95], *p* < 0.00001) was significantly higher in the bapineuzumab group, compared to the placebo group. However, the overall risk ratio did not favor either of the two groups in terms of the frequency of neoplasms (RR = 2.42, 95% CI [0.57, 10.28], *p* = 0.23), fatal adverse events (RR = 1.32, 95% CI [0.73, 2.40], *p* = 0.36), headache (RR = 1.03, 95% CI [0.81, 1.32], *p* = 0.8), vomiting (RR = 0.92, 95% CI [0.55, 1.55], *p* = 0.76), delirium (RR = 2.21, 95% CI [0.36, 13.53], *p* = 0.39), hypertension (RR = 0.49, 95% CI [0.12, 2.12], *p* = 0.34), convulsions (RR = 2.24, 95% CI [0.76, 6.58], *p* = 0.14), and falls (RR = 0.98, 95% CI [0.80, 1.21], *p* = 0.86); Fig. 5. For all adverse events, pooled studies were homogenous (Chi-Square *p* > 0.1).
III. Subgroup analysis
  A stratification analysis was performed to investigate the effect of individual doses of bapineuzumab on the clinical outcomes. All bapineuzumab doses (0.15, 0.5, 1, and 2 mg/kg) were similar to placebo in terms of change from baseline in ADAS-cog11, DAD, and MMSE scores, except for the 0.15 mg/kg dose, which caused a significant worsening on the ADAS-cog11 (MD = 5.6, 95% CI [0.22, 10.98], *p* = 0.04); Fig. 6.
  In APOE-4 carriers, bapineuzumab was significantly associated with vasogenic edema (RR = 39.36, 95% CI [9.82, 157.78], *p* < 0.00001), compared to placebo. Pooled studies [13, 16, 26] were homogenous (I^2^ = 52%, *p* = 0.12). In APOE-4 non-carriers, bapineuzumab was less significantly associated with vasogenic edema (RR = 8.45, 95% CI [1.61, 44.26], *p* = 0.01), in comparison to placebo. Pooled studies [13, 16, 26] were homogenous (I^2^ = 0%, *p* = 0.52).

## Discussion

Evaluation of the therapeutic efficacy of anti-Aβ monoclonal antibodies in AD depends primarily on cognitive and functional outcomes, while measurement of CSF biomarkers and neuroimaging techniques can only supplement these components. There has been an increasing number of phase II and phase III clinical trials in this regard over the past decade. However, this is the first systematic review and meta-analysis about the safety and efficacy of a monoclonal antibody in patients with mild to moderate AD. Our analysis showed that bapineuzumab can effectively reduce CSF phosphorylated tau protein concentration; however, this was not translated into improved clinical outcomes. Moreover, bapineuzumab significantly increased the risk of serious TEAEs and amyloid related imaging abnormalities in comparison to placebo.

Our results are consistent with the findings of a pooled analysis of two phase II clinical trials that showed that bapineuzumab can efficiently reduce CSF phosphorylated tau protein concentration [28]. This effect was more pronounced in APOE-4 carrier patients; a finding that was later confirmed in phase III trials [16]. Exploratory analysis in phase II studies showed favorable therapeutic trends on ADAS-Cog and DAD scales [13, 27]; however, phase III studies with larger sample sizes found no evidence of similar therapeutic effects [16]. The finding that the biological effect of lowering CSF phosphorylated tau protein concentration was not translated into improved clinical outcomes can be attributed to a variety of reasons. It is possible that the quantity of amyloid removal is not sufficient or that an important species of Aβ proteins was not adequately targeted. It is also possible that Aβ proteins are not the ideal target for therapeutic interventions. Because amyloid deposition in the brain starts years before the appearance of symptoms, Sperling et al. hypothesized that targeting these proteins after development of dementia may be too late to improve clinical outcomes [29]. These hypotheses need further verification to develop new treatments that can adequately target the etiology of the disease.

The failure of bapineuzumab to improve clinical outcomes in AD patients appears to be inherent to the targeted therapeutic mechanism rather than the drug itself or its route of administration. A recent study by Novak et al. showed that subcutaneous injection of bapineuzumab at doses ranging from 2 to 20 mg for 12 months failed to demonstrate a significant improvement of clinical outcomes; however, it showed a lower incidence of vasogenic edema, in comparison to studies that have utilized the intravenous route [31]. Two phase III trials have shown that solanezumab, another monoclonal antibody that targets Aβ proteins (administered intravenously at a dose of 400 mg every 4 weeks), did not affect the clinical outcomes in patients with moderate AD, but showed a potential clinical effect in mild AD [30]. We are aware of few ongoing phase III trials about gantenerumab (administered intravenously at a dose range of 60 to 200 mg every 4 weeks), which is the first fully - humanized monoclonal antibody to target both the N-terminal and central region of Aβ proteins.

The findings from our analysis indicated that bapineuzumab had no significant impact on the brain amyloid burden, measured by Pittsburgh compound B- Positron emission tomography (PIB-PET). Although this may contradict with our aforementioned finding that bapineuzumab reduces CSF p-tau protein concentration, the value of PIB-PET in diagnosis and therapeutic follow up of patients with AD is generally doubtable. Both phase III trials about bapineuzumab and solanezumab showed that around 25% of patients had negative PIB-PET scans at baseline, indicating that they did not have AD from the start [32]. Future trials are recommended to incorporate amyloid thresholds in their eligibility criteria [16].

In terms of safety, our analysis showed that bapineuzumab increases the risk of cerebral vasogenic edema. Four included studies (three reports), along with our analysis, showed that bapineuzumab-induced vasogenic edema was more frequent in APOE-4 carriers and higher dose groups [13, 16, 32]. The etiology of vasogenic edema is still obscure, but it may be related to vascular amyloid burden. The finding that amyloid deposition is more extensive in APOE-4 carriers’ cerebral blood vessels than those of non-carriers supports that theory [33]. Moreover, pre-existing cerebral amyloid angiopathy may slow drainage of interstitial fluid after mobilization of Aβ proteins [34]. However, all of these studies reported that vasogenic edema resolved on MRI upon dose adjustment or discontinuation of treatment. According to our analysis, the risk of serious TEAEs was significantly higher in bapineuzumab treated patients, compared to control patients. These disadvantages, combined with the lack of clinical benefits, stand against further clinical development of bapineuzumab.

The low risk of bias in the included double-blinded, randomized, controlled trials adds to the credibility of our evidence. A stratification analysis was performed to evaluate the efficacy of individual doses in comparison to placebo. We performed all steps in strict accordance to the Cochrane handbook of systematic reviews and reported them according to the preferred reporting items for systematic reviews and meta-analysis (PRISMA) statement guidelines.

The generalization of our results can be limited by the small number of available trials, offering a relatively small number of patients. Although we could not statistically assess for publication bias, we are aware of several related studies that were registered on clinicaltrials.gov, but were terminated before completion after the publication of the negative results of phase III trials. There were 762 (32%) discontinuations in the included studies; however, we believe this is unlikely to influence our results because all studies analyzed their data in an intention to treat approach.

Future randomized trials should investigate the safety and efficacy of combining bapineuzumab or other monoclonal antibodies with other therapeutic strategies for dementia. Further resources should be allocated to basic neuroscience research to expand the understanding of the basic pathological mechanisms of AD and target them with novel therapeutic strategies.

## Conclusions

Although bapineuzumab effectively reduced CSF phosphorylated tau protein concentration, this was not translated into improved clinical outcomes. Moreover, bapineuzumab significantly increased the risk of serious TEAEs and amyloid related imaging abnormalities. In light of the current evidence, bapineuzumab should not be used to treat patients with mild to moderate AD. Future studies should investigate the effect of combining bapineuzumab with other therapeutic strategies and reevaluate the efficacy of targeting amyloid β proteins in AD therapy.

## Additional files

Additional file 1: Shows the reasons for exclusion of retrieved articles during full text screening. (DOCX 31 kb)

Additional file 2: Shows the authors’ judgements for risk of bias assessment domains with supporting reasons. (DOCX 20 kb)

## Abbreviations

AD: Alzheimer’s disease
ADAS-Cog 11: Alzheimer disease assessment scale - Cognitive subscale 11 items
Aβ: Amyloid βeta proteins
CDR-SOB: Clinical dementia rating scale – Sum of boxes
DAD: Disability assessment for dementia
MMSE: Mini-mental state examination
PIB-PET: Pittsburg Compound B – Positron emission tomography
SVUR: Standardized uptake value ratio
TEAEs: Treatment emergent adverse events
