# Supplementary material for: Bapineuzumab for mild to moderate Alzheimer’s disease: a meta-analysis of randomized controlled trials
Source: BMC Neurol. 2017 Apr 4;17:66. doi: 10.1186/s12883-017-0850-1 (PMC5381133; doi:10.1186/s12883-017-0850-1)
Supplement: Supplementary file 1 — Shows the reasons for exclusion of retrieved articles during full text screening. (DOCX 31 kb) [file 12883_2017_850_MOESM1_ESM.docx]

**Additional file 1: Reasons for exclusion.**

| **Reason for Exclusion** | **Author, Year** |
| --- | --- |
| Pharmacokinetic analysis. | (Adedokun et al., 2013) |
| Secondary report. | (Arrighi et al., 2015) |
| Pharmacokinetic analysis. | (Bard, Fox, Friedrich, Kinney, & Yednock, 2011) |
| Pooled analysis. | (Blennow et al., 2010) |
| Post-hoc analysis of clinical trials. | (Blennow et al., 2012) |
| In vitro study. | (Bouter et al., 2015) |
| Secondary report. | (Fagan, 2012) |
| Secondary report. | (N C Fox et al., 2012) |
| Secondary report. | (Nick C Fox, DeCarli, Black, & Grundman, 2009) |
| Study protocol. | (Grundman & Black, 2008) |
| Secondary report. | (Honig et al., 2009) |
| Pharmacokinetic analysis. | (Hu, Adedokun, Ito, Raje, & Lu, 2015) |
| Pharmacokinetic analysis. | (Hutmacher et al., 2013) |
| Literature review. | (Kerchner & Boxer, 2010) |
| Literature review. | (Khorassani & Hilas, 2013) |
| Correspondence. | (Laino, 2011) |
| Correspondence. | (Laske, 2014) |
| Correspondence. | (Laskowitz & Kolls, 2010) |
| Secondary report. | (Liu et al., 2015) |
| Study abstract. | (Novak, Di, et al., 2014) |
| Study abstract. | (Novak, Einstein, et al., 2014) |
| Study abstract. | (Novak et al., 2015) |
| Literature review. | (Panza et al., 2010) |
| Literature review. | (Panza et al., 2011) |
| Pharmacokinetic analysis. | (Raskind et al., 2009) |
| Non-randomized study. | (Roher et al., 2013) |
| Literature review. | (Scheltens, Sperling, Salloway, & Fox Iv, 2012) |
| Study abstract. | (R Sperling, Salloway, Raskind, & others, 2012) |
| Secondary report. | (Reisa Sperling, Salloway, Raskind, & others, 2012) |
| Secondary report. | (Streffer et al., 2013) |

**References:**

Adedokun, O., Lu, M., Ito, K., Raje, S., Samtani, M., Xu, S., … Hu, C. (2013). Confirmatory Population Pharmacokinetic Analysis of Bapineuzumab in Subjects with Mild to Moderate Alzheimer’s Disease in Two Phase 3 Studies (ELN115727-301 and ELN115727-302) (Vol. 40, pp. S135–S136). SPRINGER/PLENUM PUBLISHERS 233 SPRING ST, NEW YORK, NY 10013 USA.

Arrighi, H. M., Barakos, J., Barkhof, F., Tampieri, D., Jack, C., Melançon, D., … Brashear, H. R. (2015). Amyloid-related imaging abnormalities-haemosiderin (ARIA-H) in patients with Alzheimer’s disease treated with bapineuzumab: a historical, prospective secondary analysis. *Journal of Neurology, Neurosurgery & Psychiatry*, jnnp–2014. Retrieved from https://jnnp.bmj.com/content/early/2015/06/12/jnnp-2014-309493.full

Bard, F., Fox, M., Friedrich, S., Kinney, G., & Yednock, T. (2011). Unique brain PK properties of 3D6 and bapineuzumab depend on cerebral amyloid load in PDAPP transgenic mice. *Alzheimer’s & Dementia*, *7*(4), e54.

Blennow, K., Zetterberg, H., Rinne, J. O., Salloway, S., Wei, J., Black, R., … others. (2012). Effect of immunotherapy with bapineuzumab on cerebrospinal fluid biomarker levels in patients with mild to moderate Alzheimer disease. *Arch Neurol*, *69*(8), 1002–1010. Retrieved from http://archotol.jamanetwork.com/data/Journals/NEUR/24759/noc120004_1002_1010.pdf

Blennow, K., Zetterberg, H., Wei, J., Liu, E., Black, R., & Grundman, M. (2010). Immunotherapy with bapineuzumab lowers CSF tau protein levels in patients with Alzheimer’s disease. *Alzheimer’s & Dementia*, *6*(4), S134–S135.

Bouter, Y., Noguerola, J. S. L., Tucholla, P., Crespi, G. A. N., Parker, M. W., Wiltfang, J., … Bayer, T. A. (2015). Abeta targets of the biosimilar antibodies of Bapineuzumab, Crenezumab, Solanezumab in comparison to an antibody against N-truncated Abeta in sporadic Alzheimer disease cases and mouse models. *Acta Neuropathol*, *130*(5), 713–729. Retrieved from http://link.springer.com/article/10.1007/s00401-015-1489-x

Fagan, T. (2012). Bapineuzumab phase 3: target engagement, but no benefit.

Fox, N C, Salloway, S., Sperling, R., Raskind, M., Ferris, S., Honig, L. S., & others. (2012). Bapineuzumab Phase 3 trials in mild to moderate Alzheimer’s disease dementia in apolipoproteinE e4 carriers (Study 302) and non-carriers (Study 301): CSF and Volumetric MRI Biomarkers. *Clinical Trials in Alzheimer’s Disease. Available at: http://www. ctad. fr/07-download/Congres2012/PressRelease/Final-Fox-CTAD-Presentation-10-29-12. pdf. Accessed*, *27*.

Fox, Nick C, DeCarli, C., Black, R., & Grundman, M. (2009). Effect of Bapineuzumab on MRI Measures of Cerebral Volume Change in Patients with Alzheimer’s Disease (Vol. 72, pp. A147–A147). LIPPINCOTT WILLIAMS & WILKINS 530 WALNUT ST, PHILADELPHIA, PA 19106-3621 USA.

Grundman, M., & Black, R. (2008). Clinical trials of bapineuzumab, a beta-amyloid-targeted immunotherapy in patients with mild to moderate Alzheimer’s disease. *Alzheimer’s & Dementia*, *4*(4), T166.

Honig, L. S., Gilman, S., Morris, K., Black, R., Grundman, M., & Francis, G. (2009). Safety Profile of Bapineuzumab in a Phase II Trial of Mild-to-Moderate Alzheimer’s Disease (AD) (Vol. 72, pp. A272–A272). LIPPINCOTT WILLIAMS & WILKINS 530 WALNUT ST, PHILADELPHIA, PA 19106-3621 USA.

Hu, C., Adedokun, O., Ito, K., Raje, S., & Lu, M. (2015). Confirmatory population pharmacokinetic analysis for bapineuzumab phase 3 studies in patients with mild to moderate Alzheimer’s disease. *The Journal of Clinical Pharmacology*, *55*(2), 221–229. Retrieved from http://onlinelibrary.wiley.com/doi/10.1002/jcph.393/full

Hutmacher, M., Hu, C., Guenzler-Pukall, V., Arrighi, M., Ito, K., Samtani, M., … Lu, M. (2013). Pharmacokinetic-Pharmacodynamic Modeling of Amyloid-Related Imaging Abnormalities of Edema Following Intravenous Administration of Bapineuzumab to Subjects with Mild to Moderate Alzheimer’s Disease (Vol. 40, pp. S137–S138). SPRINGER/PLENUM PUBLISHERS 233 SPRING ST, NEW YORK, NY 10013 USA.

Kerchner, G. A., & Boxer, A. L. (2010). Bapineuzumab. *Expert Opin Biol Ther*, *10*(7), 1121–1130. Retrieved from http://www.tandfonline.com/doi/abs/10.1517/14712598.2010.493872

Khorassani, F., & Hilas, O. (2013). Bapineuzumab, an investigational agent for Alzheimer’s disease. *PT*, *38*(2), 89–91. Retrieved from https://www.researchgate.net/profile/Olga_Hilas/publication/236228733_Bapineuzumab_an_Investigational_Agent_For_Alzheimer’s_Disease/links/542c141b0cf29bbc126b2f1b.pdf

Laino, C. (2011). Cerebral Edema Common, but Found to be Manageable, with Bapineuzumab. *Neurology Today*, *11*(16), 26–29. Retrieved from http://journals.lww.com/neurotodayonline/Citation/2011/08180/Cerebral_Edema_Common,_but_Found_to_be_Manageable,.10.aspx

Laske, C. (2014). Phase 3 trials of solanezumab and bapineuzumab for Alzheimer’s disease. *N Engl J Med*, *370*(15), 1459. Retrieved from http://www.nejm.org/doi/full/10.1056/NEJMc1402193

Laskowitz, D. T., & Kolls, B. J. (2010). A phase 2 multiple ascending dose trial of bapineuzumab in mild to moderate Alzheimer disease. *Neurology*, *74*(24), 2026–2027. Retrieved from http://www.neurology.org/content/74/24/2026.short

Liu, E., Schmidt, M. E., Margolin, R., Sperling, R., Koeppe, R., Mason, N. S., … others. (2015). Amyloid-β 11C-PiB-PET imaging results from 2 randomized bapineuzumab phase 3 AD trials. *Neurology*, *85*(8), 692–700. Retrieved from http://www.neurology.org/content/85/8/692.short

Novak, G., Di, J., Brashear, R. H., Werth, J., Booth, K., Margolin, R., … Liu, E. (2014). Efficacy and safety of subcutaneous Bapineuzumab. *Alzheimer’s & Dementia: The Journal of the Alzheimer's Association*, *10*(4), P446–P447. Retrieved from http://www.alzheimersanddementia.com/article/S1552-5260(14)01251-5/abstract

Novak, G., Einstein, S. G., Bracoud, L., Pachai, C., Schaerer, J., Brashear, R. H., … Liu, E. (2014). Rates of change in brain volume with subcutaneous bapineuzumab. *Alzheimer’s & Dementia: The Journal of the Alzheimer's Association*, *10*(4), P25–P26. Retrieved from http://www.alzheimersanddementia.com/article/S1552-5260(14)00693-1/abstract

Novak, G., Fox, N., Clegg, S., Nielsen, C., Einstein, S., Lu, Y., … others. (2015). Changes in Brain Volume with Bapineuzumab in Mild to Moderate Alzheimer’s Disease. *Journal of Alzheimer’s Disease*, (Preprint), 1–12. Retrieved from http://content.iospress.com/articles/journal-of-alzheimers-disease/jad150448

Panza, F., Frisardi, V., Imbimbo, B. P., D’Onofrio, G., Pietrarossa, G., Seripa, D., … Solfrizzi, V. (2010). Bapineuzumab: anti-β-amyloid monoclonal antibodies for the treatment of Alzheimer’s disease. *Immunotherapy*, *2*(6), 767–782. Retrieved from http://www.futuremedicine.com/doi/abs/10.2217/imt.10.80

Panza, F., Frisardi, V., P Imbimbo, B., Seripa, D., Paris, F., Santamato, A., … Solfrizzi, V. (2011). Anti-β-amyloid immunotherapy for Alzheimer’s disease: focus on bapineuzumab. *Curr Alzheimer Res*, *8*(8), 808–817. Retrieved from http://www.ingentaconnect.com/content/ben/car/2011/00000008/00000008/art00001

Raskind, M., Liang, E., Sperling, R., Boxer, A., Ross, J., Brody, M., … Grundman, M. (2009). Pharmacokinetics and pharmacodynamics of bapineuzumab following multiple intravenous infusions in patients with mild-to-moderate Alzheimer’s disease. *Alzheimer’s & Dementia*, *5*(4), P415–P416.

Roher, A. E., Cribbs, D. H., Kim, R. C., Maarouf, C. L., Whiteside, C. M., Kokjohn, T. A., … others. (2013). Bapineuzumab alters aβ composition: implications for the amyloid cascade hypothesis and anti-amyloid immunotherapy. *PLoS One*, *8*(3), e59735. Retrieved from http://journals.plos.org/plosone/article?id=10.1371/journal.pone.0059735

Scheltens, P., Sperling, R., Salloway, S., & Fox Iv, N. (2012). Bapineuzumab IV phase 3 results. *J Nutr Health Aging*, *16*(9), 795–872.

Sperling, R, Salloway, S., Raskind, M., & others. (2012). A randomized, double-blind, placebocontrolled clinical trial of intravenous bapineuzumab in patients with mild to moderate Alzheimer’s disease who are apolipoprotein e ε4 carriers. Retrieved from http://www2.kenes.com/efns/info/Documents/Sperling_Bapineuzumab IV Study 302_EFNS Presentation Slides_9-11-2012.pdf

Sperling, Reisa, Salloway, S., Raskind, M., & others. (2012). Bapineuzumab phase 3 trials in mild to moderate Alzheimer’s disease dementia in apolipoprotein E ε4 carriers (Study 302) and non-carriers (Study 301). Retrieved from http://www.ctad-alzheimer.com/sites/ctad.prod/files/files/Archives Press/Final-Sperling-CTAD-Presentation-10-29-12.pdf

Streffer, J., Blennow, K., Salloway, S., Zetterberg, H., Xu, Y.-Z., Lu, Y., … others. (2013). Effect of bapineuzumab on CSF p-tau and t-tau in mild-to-moderate Alzheimer’s disease: Results from two phase III trials in APOE-ε4 carriers and noncarriers. *Alzheimer’s & Dementia: The Journal of the Alzheimer's Association*, *9*(4), P138. Retrieved from http://www.alzheimersanddementia.com/article/S1552-5260(13)00231-8/fulltext?mobileUi=0
