# Supplementary material for: Bapineuzumab for mild to moderate Alzheimer’s disease: a meta-analysis of randomized controlled trials
Source: BMC Neurol. 2017 Apr 4;17:66. doi: 10.1186/s12883-017-0850-1 (PMC5381133; doi:10.1186/s12883-017-0850-1)
Supplement: Supplementary file 2 — Shows the authors’ judgements for risk of bias assessment domains with supporting reasons. (DOCX 20 kb) [file 12883_2017_850_MOESM2_ESM.docx]

**Additional file 2: Risk of Bias Assessment of included studies**

| **Salloway et al. Carrier 2014** | **Risk of Bias** | **Reason/Quotations** |
| --- | --- | --- |
| Random sequence generation (selection bias) | Low risk | "1000 participants, who would be randomly assigned in a 3:2 ratio". |
| Allocation concealment (selection bias) | Unclear risk |  |
| Blinding of participants and personnel (performance bias) | Low risk | "Double-blind, randomized, placebo-controlled, phase 3 trials". |
| Blinding of outcome assessment (detection bias) | Low risk | "Double-blind, randomized, placebo-controlled, phase 3 trials". |
| Incomplete outcome data (attrition bias) | Low risk | "The modified intention-to-treat population included participants who received at least one dose of a study drug and underwent evaluation of the co-primary efficacy end points at baseline and at least once after baseline". |
| Selective reporting (reporting bias) | Low risk | Protocol not available but it is expected that all major outcomes were reported. |
| Other bias | Unclear risk |  |
| **Salloway et al. Non Carrier 2014** | **Risk of Bias** | **Reason/Quotations** |
| Random sequence generation (selection bias) | Low risk | "1300 participants, who would be randomly assigned in a 3:3:4 ratio". |
| Allocation concealment (selection bias) | Unclear risk |  |
| Blinding of participants and personnel (performance bias) | Low risk | "Double-blind, randomized, placebo-controlled, phase 3 trials". |
| Blinding of outcome assessment (detection bias) | Low risk | "Double-blind, randomized, placebo-controlled, phase 3 trials". |
| Incomplete outcome data (attrition bias) | Low risk | "The modified intention-to-treat population included participants who received at least one dose of a study drug and underwent evaluation of the co-primary efficacy end points at baseline and at least once after baseline". |
| Selective reporting (reporting bias) | Low risk | Protocol not available but it is expected that all major outcomes were reported. |
| Other bias | Unclear risk |  |
| **Salloway et al. 2009 phase II** | **Risk of Bias** | **Reason/Quotations** |
| Random sequence generation (selection bias) | Low risk | "234 patients were randomly assigned to receive either IV bapineuzumab or placebo, in a 8:7 ratio". |
| Allocation concealment (selection bias) | Unclear risk |  |
| Blinding of participants and personnel (performance bias) | Low risk | "Phase 2, multicenter, randomized, double-blind, placebo-controlled, multiple ascending dose study". |
| Blinding of outcome assessment (detection bias) | Low risk | "Phase 2, multicenter, randomized, double-blind, placebo-controlled, multiple ascending dose study". |
| Incomplete outcome data (attrition bias) | Low risk | "The modified intent-to-treat (mITT) population included patients who received at least one dose of study drug and had one or more post-baseline, co-primary efficacy evaluations". |
| Selective reporting (reporting bias) | Low risk | Protocol not available but it is expected that all major outcomes were reported. |
| Other bias | Unclear risk |  |
| **Arai et al. 2016** | **Risk of Bias** | **Reason/Quotations** |
| Random sequence generation (selection bias) | Low risk | "Eligible participants were randomly assigned to receive one infusion of either bapineuzumab 0.15, 0.5, 1.0 or 2.0 mg/kg, or a placebo". |
| Allocation concealment (selection bias) | Unclear risk |  |
| Blinding of participants and personnel (performance bias) | Low risk | "Phase 1, multicenter, randomized, double-blind, placebo-controlled, single ascending-dose study". |
| Blinding of outcome assessment (detection bias) | Low risk | "Phase 1, multicenter, randomized, double-blind, placebo-controlled, single ascending-dose study". |
| Incomplete outcome data (attrition bias) | Low risk | All patients who were randomized and received study drug were included in the study analysis. |
| Selective reporting (reporting bias) | Low risk | Protocol not available but it is expected that all major outcomes were reported. |
| Other bias | Unclear risk |  |
| **Black et al. 2010** | **Risk of Bias** | **Reason/Quotations** |
| Random sequence generation (selection bias) | Low risk | "Patients were randomly assigned in a blinded fashion (ie, third-party un-blinded) to receive study drug". |
| Allocation concealment (selection bias) | Unclear risk |  |
| Blinding of participants and personnel (performance bias) | Low risk | "Multicenter, randomized, third-party unblinded, placebo-controlled, single dose study". |
| Blinding of outcome assessment (detection bias) | Low risk | "Multicenter, randomized, third-party unblinded, placebo-controlled, single dose study". |
| Incomplete outcome data (attrition bias) | Low risk | All patients who were randomized and received study drug were included in the study analysis. |
| Selective reporting (reporting bias) | Low risk | Protocol not available but it is expected that all major outcomes were reported. |
| Other bias | Unclear risk |  |
| **Rinne et al. 2010** | **Risk of Bias** | **Reason/Quotations** |
| Random sequence generation (selection bias) | Low risk | "Phase 2, multicenter, randomised, double-blind, placebo-controlled, ascending-dose study". |
| Allocation concealment (selection bias) | Low risk | "Randomisation was by interactive voice response system; masking was achieved with numbered kit allocation". |
| Blinding of participants and personnel (performance bias) | Low risk | "During the study, patients, investigators (both image analysts and clinical assessors), study site personnel, and sponsor staff were masked to treatment". |
| Blinding of outcome assessment (detection bias) | Low risk | "During the study, patients, investigators (both image analysts and clinical assessors), study site personnel, and sponsor staff were masked to treatment". |
| Incomplete outcome data (attrition bias) | Low risk | "All patients who received at least one dose of study drug and had one or more post-baseline were included in the modified intent-to-treat (mITT) population". |
| Selective reporting (reporting bias) | Low risk | Protocol not available but it is expected that all major outcomes were reported. |
| Other bias | Unclear risk |  |
